# Supplementary figures and images for: Image-quality metric system for color filter array evaluation
Source: PLoS One. 2020 May 11;15(5):e0232583. doi: 10.1371/journal.pone.0232583 (PMC7213733; doi:10.1371/journal.pone.0232583)

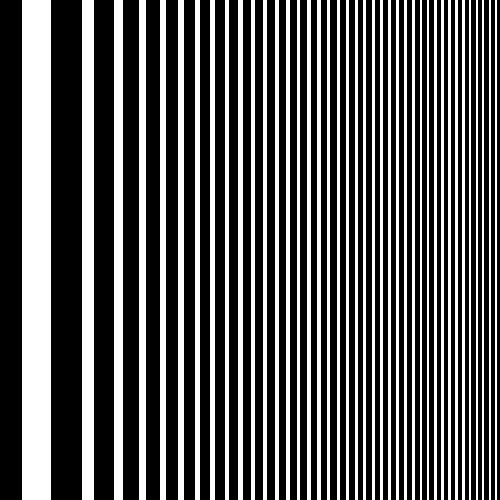

Supplement: S1 Fig — (JPG) [file pone.0232583.s002.jpg]

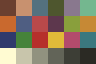

Supplement: S2 Fig — (TIF) [file pone.0232583.s003.tif]
