# Supplementary material for: Image-quality metric system for color filter array evaluation
Source: PLoS One. 2020 May 11;15(5):e0232583. doi: 10.1371/journal.pone.0232583 (PMC7213733; doi:10.1371/journal.pone.0232583)
Supplement: Attachment — Submitted filename: Response to Reviewers_2.docx [file pone.0232583.s007.docx]

Reviewer #2: The article presents that: currently the most frequently used method of demosaicing in terms of cost and time of computation is bilinear interpolation. On what basis did it say so? There is, for example, the method of the nearest neighbour interpolation which is even faster and requires much less resources.

Ans) Yes, right. The description has been deleted.

It would be worthwhile to see how the results obtained for different CFA systems behave for different demosaicing methods.

Ans) We did experiments with Laplacian, Adaptive laplcian, and POCS demosaicing. The following has been added to the experiment.

-------------------------------------------------------------------------------------------------------------

Table 1 shows the results of each of the metrics for test CFAs using different demosaicing methods. The red boxes in the table represent the highest scoring CFAs in the demosaicing and metrics. RGB4 CFA for bilinear demosaicing, RGBW1 CFA for laplacian demosaicing, Bayer CFA for adaptive laplacian demosaicing, and RGB5 CFA for POCS demosaicing have the highest scores. Also, regardless of demosaicing methods, Bayer, RGB1~RGB5, CMY1~CMY3, and RGBW1~RGBW3 CFA show seven, two, zero, one, three, three, one, one, four, seven, two, and two high score metrics respectively.

**Table 1.** Comparison results of all metrics for test CFAs according to demosaicing methods.

**Figure 19.** Polar coordinate visualization using proposed CFA image quality evaluation system for test CFAs (Magenta dotted line : Bilinear, Cyon dotted line : Laplacian, Green dotted line : Adaptive Laplacian, Blue dotted line : POCS, and Red solid line : Average).

The evaluation results by all metrics for the test CFAs are shown in Figure 19 and Table 2. Figure 19 provides a polar coordinate visualization through the proposed CFA image-quality evaluation system for the test CFAs. It can be seen that the performance of the Test CFAs can be easily visualized. Table 2 shows the performance comparison of test CFAs by the normalized metrics mentioned in section 2.2.9. Bayer CFA for adaptive laplacian demosaicing and RGBW1 CFA for Bilinear, Laplacian, and POCS demosacing have the best performance. The ranking of the test CFAs by the demosaicing methods and metrics used in this paper is as follows: RGBW1, Bayer, RGBW3, RGBW2, RGB5, RGB2, RGB3, CMY2, RGB1, RGB4, CMY1 and CMY3.

**Table 2.** Performance comparison of test CFAs by normalized metrics.

Consideration should be given to the statement: The pipeline typically consists of demosaicking, noise reduction, white balance, CFA interpolation, color conversion, and gamma correction for rendering the sensor data. Is the formulation correct and is this the correct order of operations?

Ans) Yes, right. Figure 2 was corrected as the following.

**Figure 2.** Proposed CFA image-quality evaluation system.

The proposed quality assessment system for different CFA has been reduced to a selection of several metrics used to assess the quality of a digital image where a reference image is required. The article proposes visualization in the form of polar coordinates. For different CFA systems we obtain a different distribution, however, it is the subjective observer who must assess which of the measurements is more important for him. The authors should look at another measure of image quality assessment closely correlated with HVS. Examples of such metrics are: DSCSI, MDSI, HPSI etc… Lee, Dohyoung, and Konstantinos N. Plataniotis. "Towards a full-reference quality assessment for color images using directional statistics." IEEE Transactions on image processing 24.11 (2015): 3950-3965.

Nafchi, Hossein Ziaei, et al. "Mean deviation similarity index: Efficient and reliable full-reference image quality evaluator." IEEE Access 4 (2016): 5579-5590.

Reisenhofer, Rafael, et al. "A Haar wavelet-based perceptual similarity index for image quality assessment." Signal Processing: Image Communication 61 (2018): 33-43.

It is also worthwhile to examine the articles:

Frackiewicz, Mariusz, and Henryk Palus. "Toward a perceptual image quality assessment of color quantized images." Tenth International Conference on Machine Vision (ICMV 2017). Vol. 10696. International Society for Optics and Photonics, 2018.

Frackiewicz, Mariusz, and Henryk Palus. "New image quality metric used for the assessment of color quantization algorithms." Ninth International Conference on Machine Vision (ICMV 2016). Vol. 10341. International Society for Optics and Photonics, 2017.

Ans) Thank you for the advice. We have added MDSI and HaarPSI metric in section 2.2.7, 2.2.8, and experiment result as the following.

2.2.7. Structural and Color Distortion Using Gradient Similarity and Chromaticity Similarity

The mean deviation similarity index (MDSI) utilizes gradient and chrominance features to measure structural and color distortion [46]. A gradient-chromaticity similarity map is made by combining these two similarity maps. First, for reference (ideal) and distorted (CFA) images, *R* and *D*, gradient similarity (GS) is obtained by

| $\text{GS}(\text{x})=\frac{2G_{R}(x)G_{D}(x)+C_{1}}{G_{R}^{2}(x)+G_{D}^{2}(x)+C_{1}}$, | (7) |
| --- | --- |

Using an averaging image *F* of the *R* and *D*, two extra GS maps are calculated by

| $\text{GS}_{RF}(\text{x})=\frac{2G_{R}(x)G_{F}(x)+C_{2}}{G_{R}^{2}(x)+G_{F}^{2}(x)+C_{2}}$, | (8) |
| --- | --- |
| $\text{GS}_{DF}(\text{x})=\frac{2G_{D}(x)G_{F}(x)+C_{2}}{G_{D}^{2}(x)+G_{F}^{2}(x)+C_{2}}$, | (9) |

The enhanced gradient similarity (GS) presented in [46] is obtained by

| $\overline{\text{GS}}(\text{x})=\text{GS}(\text{x})+[\text{GS}_{DF}(\text{x})-\text{GS}_{RF}(\text{x})]$, | (10) |
| --- | --- |
| And the enhanced color similarity (CS) is obtained by |  |
| $\overline{\text{CS}}(\text{x})=\frac{2\left(H_{R}(x)H_{D}(x)+M_{R}(x)M_{D}(x)\right)+C_{3}}{H_{R}^{2}(x)+H_{D}^{2}(x)+M_{R}^{2}(x)+M_{D}^{2}(x)+C_{3}}$, | (11) |

Finally, the gradient-color similarity (GCS) is calculated as the follows:

| $\overline{\text{GCS}}(\text{x})=\alpha\overline{\text{GS}}(\text{x})+(1-\alpha)\overline{\text{CS}}(\text{x})$, | (12) |
| --- | --- |

And the MDSI is defined as the follows:

| $\text{MDSI}=\left[\frac{1}{4}\sum_{i=1}^{N} \left\vert\overline{\text{GCS}}_{i}^{1/4}-\left(\frac{1}{N}\sum_{i=1}^{N} \overline{\text{GCS}}_{i}^{1/4}\right)\right\vert\right]^{1/4}$, | (13) |
| --- | --- |

2.2.8. Perceptual Similarity Using Haar Wavelet Decomposition

The Haar wavelet-based perceptual similarity index (HaarPSI) was presented for yielding full reference image quality assessments [47]. The HaarPSI evaluates local similarities as well as entire similarities between two images by using the coefficients obtained from a Haar wavelet decomposition. For two grayscale images f_1_, f_2_, the local similarity is computed based on a two-dimensional discrete Haar wavelet transform as the following;

| $\text{HS}_{\text{f}_{1},\text{f}_{2}}^{(\text{k})}[x]=l_{\alpha}\left(\frac{1}{2}\sum_{j=1}^{2} \text{S}\left(\left\vert\left(g_{j}^{(\text{k})}*\text{f}_{1}\right)[x]\right\vert,\left\vert\left(g_{j}^{(\text{k})}*\text{f}_{2}\right)[x]\right\vert,C\right)\right),$, | (14) |
| --- | --- |

where C>0, k∈{1,2} selects either horizontal or vertical Haar wavelet filters and S represents the similarity. And a weight map which is considered in the HaarPSI is obtained by

| $\text{W}_{\text{f}}^{(\text{k})}[x]=\left\vert\left(g_{3}^{(\text{k})}*f\right)[x]\right\vert$, | (15) |
| --- | --- |

Finally, the HaarPSI is computed by

| $\text{HaarPSI}_{\text{f}_{1}\text{,f}_{2}}=l_{\alpha}^{-1}\left(\frac{\sum_{x} \sum_{k=1}^{2} HS_{\text{f}_{1}\text{,f}_{2}}^{(k)}[x]\cdot W_{\text{f}_{1}\text{,f}_{2}}^{(k)}[x]}{\sum_{x} \sum_{k=1}^{2} W_{\text{f}_{1}\text{,f}_{2}}^{(k)}[x]}\right)^{2},$, | (16) |
| --- | --- |

**Figure 18.** MDSI and HaarPSI measurement results for test CFAs.

Figure 18 shows the MDSI and HaarPSI measure for the test CFAs. MDSI means that the closer to 0, the higher the structural and color similarity. All test CFAs show relatively good MDSI values. The closer the GCS is to 1, the higher the structural and color similarity between the two images. Most CFAs have a low GCS near indigo (or blue), which means that similarity is low in that area. In perceptual similarity comparison, RGB-based CFAs show relatively good HaarPSI values, while CMY or RGBW-based CFAs have rather low HaarPSI values.
