# Supplementary material for: Image-quality metric system for color filter array evaluation
Source: PLoS One. 2020 May 11;15(5):e0232583. doi: 10.1371/journal.pone.0232583 (PMC7213733; doi:10.1371/journal.pone.0232583)
Supplement: Attachment — Submitted filename: Response to Reviewers_2(R2).docx [file pone.0232583.s008.docx]

Reviewer #2: Are there statistically significant differences between the CFA systems tested for quality indices?

Sol) We can see that the metric score difference between the worst and the best CFA for each demosaicing method is significant. A quantitative analysis was conducted and Figure 17 was added as follows.

-------------------------------------------------------------------------------------------------------------

(Line 490) Bayer shows the best performance for adaptive laplacian demosaicing and obtained higher metric scores of 0.0203~0.1228 compared to the other test CFAs. On the other hand, RGBW1 shows the best performance for bilinear, laplacian, and POCS demosacing and received superior metric socres of 0.0026~0.2323, 0.003~0.1014, 0.0084~0.1611 for the respective demosaicing methods. For all the demosaicing methods used in this paper, the best CFA was RGBW1 and acquired higher scores of 0.0228 to 0.1081 compared to the other test CFAs. Based on the analysis in Table 3, the CFA ranks for the respective and total demosaicing methods are shown in Figure 17. We can see that the metric score difference between the worst and the best CFA for each demosaicing method is significant.

Figure 17. CFA rank for (a) Blinear, (b) Laplacian, (c) Adaptive laplacian, (d) POCS, and (e) Total demosaicing.

In Table 1 there are identical values for HPSI, MDSI and others for different CFA systems, maybe you need to use a different way of presenting the results?

Sol) The analysis was conducted as follows.

----------------------------------------------------------------------------------------------------------

(Line 468) MDSI values are similar for each CFA, however the GCS distribution is different (especially in the red and blue color regions). Based on this phenomenon, it can be deduced that MDSI value may vary according to color distribution of the input image. For each test CFAs, HaarPSI ranges 0.60 to 0.75. Total images rendered show entirely high HaarPSI value because the degree of distortion is weak compared to the original image.

<I appreciate for your precious comments !!>
